# A robust, hand-powered, instrument-free sample preparation system for point-of-care pathogen detection

**DOI:** 10.1038/s41598-019-52922-y

**Published:** 2019-11-08

**Authors:** Fei Zhao, Eun Yeong Lee, Geun Su Noh, Jaehyup Shin, Huifang Liu, Zhen Qiao, Yong Shin

**Affiliations:** 1 Department of Convergence Medicine, Asan Medical Institute of Convergence Science and Technology, Asan Medical Center, University of Ulsan College of Medicine, Seoul, Republic of Korea; 2 Academy of Medical Engineering and Translational Medicine, Tianjin University, Tianjin Key Laboratory of Brain Science and Neural Engineering, Tianjin, China

**Keywords:** Solid-phase microextraction, Nanoparticles

## Abstract

Here, we describe a simple, universal protocol for use in nucleic acid testing-based pathogen diagnostics, which requires only hand-powered sample preparation, including the processes of pathogen enrichment and nucleic acid isolation. The protocol uses low-cost amine-functionalized diatomaceous earth with a 1-μm Teflon filter as a reaction matrix in both stages of the process, using homobifunctional imidoesters. Using a simple syringe as a pump, the capture efficiency for a large sample volume (<50 mL) was enhanced by up to 98.3%, and the detection limit was 1 CFU/mL, 100-fold better than that of common commercial nucleic acid isolation kit. This protocol can also be combined with commercialized 96-well filter plates for robust sample preparation. Our proposed system is robust, simple, low-cost, universal, and rapid (taking <20 min), and it works regardless of the ambient environment and sample pretreatment, requiring no electricity or instruments. Its benefits include the simplicity of producing its components and its ease of operation, and it can be readily integrated with other assays for point-of-care diagnostics.

## Introduction

The diagnosis of pathogens is a critical issue in global healthcare, especially in resource-limited settings with little or no access to sophisticated laboratory techniques^1^. Various methods have been developed for diagnosing pathogenic diseases, including blood culture^2^, blood chemistry^3^, flow cytometry^4^, immunoassays^5^, and nucleic acid testing (NAT)^6^. Although bacterial blood cultures are widely used for clinical diagnosis, this approach is time-consuming (usually taking several days), expensive, uses species-specific protocols, and requires laboratory facilities^7^. As an alternative, NAT has been the subject of growing interest; its protocols are relatively fast and universal and it is amenable to various applications for diagnostic testing, including window period diagnosis, immunovariant virus detection, and immunosilent/occult infection identification^8–10^. However, existing NAT-based assays are generally complex both for commercially available products and for laboratory-developed assays^11^. They generally involve intricate pretreatments (such as pre-fabrication or sample centrifugation), which require laboratory-based procedures involving multiple steps, skilled technicians, and specific instruments^12^. These drawbacks limit the application of NAT to point-of-care testing (POCT), especially in resource-limited settings.

There are three main aspects to NAT-based POCT: sample preparation, template amplification, and signal detection^13^. Efforts to improve NAT-based POCT assays have been directed at developing simple and effective amplification and detection methods; few researchers have focused on the initial sample preparation step^14^. Numerous extraction-free NAT-based POCT assays have been developed in which DNA templates are directly amplified in crude samples without the need for upstream isolation; however, these are still limited by low sensitivity and high cost^15–17^. Sample preparation, including nucleic acid (NA) isolation from biological matrices and chemical inhibitor elimination, is critical because high-quality NA is the foundation for all of the downstream analyses^11^. In particular, it is necessary for sample preparation to be adaptable to an open environment rather than restricted to a laboratory to eliminate the need for clean rooms in resource-limited settings^12^.

High-throughput, centralized, laboratory-based diagnosis is not practical in resource-limited settings, not only because of the high cost of permanent integrated facilities, but also owing to the limited availability of trained analysts^18^. Thus, disposable POCT assay systems appear to be a solution for such situations, as recognized in the Gates Foundation’s Global Grand Challenges^19^. For the past decade, lateral flow or immune-chromatographic strip (ICS) tests have been successfully employed as diagnostic assays in resource-limited settings^20^. A few types of ICS for certain diagnoses, such as for blood glucose monitoring, have become relatively affordable and are stable at ambient temperatures, which facilitates their packaging and transportation. However, ICS tests are commonly limited by their need for complex sample analysis^21^.

Various methods have been developed for sample preparation^12^. Among these, particle-based assays have attracted particular attention because of their high performance as sorbents and their applicability to various techniques. However, particle-based assays have both advantages and limitations. For example, the highest extraction efficiencies for NA isolation are obtained using chaotropic agents with a silica matrix, but residuals of these chaotropic agents also act as inhibitors in the NA amplification process^22^. Despite the development of several improved assays, including the use of porous polymers or magnetic functionalization^23^, it remains a challenge to develop assays that are optimized for use in resource-limited settings because none of the existing assays can easily be adapted to a POCT system. Besides, the fast technology analysis (FTA) cards provide an alternative solution as NA detection assays, which allow cost-effective, accurate, and reliable pathogen diagnosis^24^. Various paper-based assays have been developed to simplify the NA isolation process, as well as sample separation^25^, and NA storage and transformation^26^. When using conventional FTA card-based assays (including commercial kits, e.g., Whatman FTA cards), there are difficulties when faced with low-concentration analytes (femtomolar or nanomolar) and small volumes (1–1000 µL) within complex biological media (e.g., whole blood samples)^27^. However, they are not offered to the large-volume sample treatment by the intrinsic limitations, such as spotting-based sample loading process and small sample loading disk.

Assays for POCT should be optimized so that they are simple (requiring minimal facilities for product fabrication and use, and minimal personnel training), rapid, stable for logistics and storage, and low-cost. Cost is particularly important because, according to World Health Organization (WHO) guidelines, cost minimization is one of the main requirements for diagnostics in resource-limited settings^28^. The lack of reliable low-cost POCTs is still considered by WHO to be a major barrier for the global control and prevention of sexually transmitted infections^29^. In addition, it is challenging to adapt assays to common clinical samples, such as of urine, blood, and sputum, because such samples often contain polymerase chain reaction (PCR) inhibitors. Thus, there is a need for an adaptable solution that integrates pathogen enrichment and NA sample preparation, which are both required for the clinical application of POCTs.

To address these challenges, we previously developed a universal protocol with instruments that could be easily integrated into other assays^30,31^. Here, we describe a hand-powered, instrument-free sample preparation system for pathogen diagnostics, intended to simplify the entire process from sample collection to high-quality NAs. This system benefits from the use of a syringe-based hand-powered design, and the assay is suitable for on-site analysis of large-volume samples without the need for any external devices. In the system, pathogens are trapped on a syringe filter in the presence of amine-functionalized diatomaceous earth (ADE) and homobifunctional imidoesters (HIs). NA samples are then isolated from the filter without any extra equipment. Using this approach, high-quality NA samples for downstream diagnosis can be prepared at room temperature within 20 min without the need for specialized equipment or centralized laboratories. It has been reported that positively charged ADE can enrich negatively charged pathogens in ordinary aqueous samples^31^; however, our earlier system was designed for DNA isolation only and was not suitable for large sample volumes. In the present study, we developed a universal protocol applicable to both DNA and RNA isolation, which could be used for large sample volumes. In the presence of HIs, which react with the amine groups of ADE, more positively charged groups are formed, resulting in enhanced pathogen enrichment^32^. In this way, negatively charged pathogens can be directly and rapidly absorbed onto the diatomaceous earth (DE) from small sample volumes (1 mL) or absorbed with only a short incubation for large sample volumes (50 mL). Furthermore, the HIs can reversibly crosslink amine groups on NAs and proteins, as well as NAs to the DE^33^. This reaction can be reversed by changing the pH, so the preparation of NA samples for diagnostics can easily be achieved by the injection of different buffers. For these reasons, this method is highly suitable for adoption into a cost-effective NA diagnostic process for various clinical samples. This hand-held assay has a number of advantages: (1) it enriches pathogens effectively and isolates NA for subsequent downstream diagnostic analysis; (2) it simplifies procedures of test manufacturing and operation; (3) the total operation time can be as little as 20 min; (4) it does not require a laboratory facility; (5) it can form the basis of a universal protocol for the preparation of various clinical samples; (6) it is inexpensive; and (7) it is robust.

## Results and Discussion

### Development of the all-in-one hand-held extraction method

**Figure 1:**
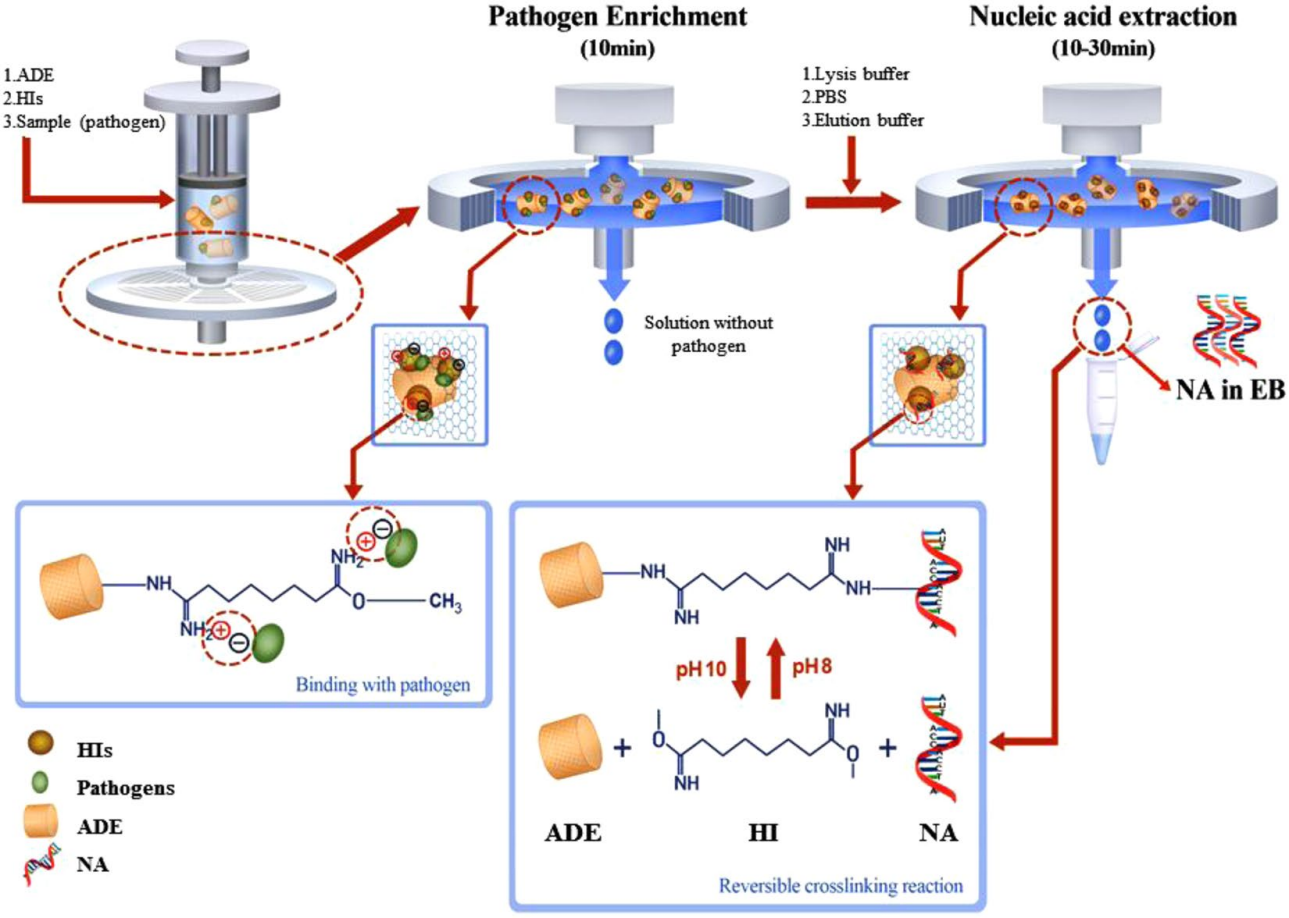
Schematic diagram illustrating nucleic acid (NA) isolation using the hand-held syringe filter method based on amine-functionalized diatomaceous earth (ADE) in conjunction of homobifunctional imidoesters (HIs; DMA, DMS, DMP). Pathogen enrichment (top middle) and NA extraction (top right) can be completed with this hand-held system without the use of any chaotropic agents or instruments within as little as 20 min for 1 mL samples and 40 min for 50 mL samples. Shown by the dashed line from the pathogen enrichment process, the pathogens are absorbed onto the surface of the ADE; this is assisted by DMS via its positively charged amidine bonds, which can interact electrostatically with negatively charged pathogens (bottom left). The principle of NA isolation is shown by the dashed lines from the NA extraction process: DMS crosslinks the pathogen NA to ADE via a covalent bond in the reaction buffer (at pH 8); this can be broken by injecting a high-pH elution buffer (pH 10) into the filter, allowing the NA to be collected (bottom right).

The assay used to collect NA templates from the various types of samples is based on an ADE modified commercial PTFE filter in combination with a syringe (Fig. 1, top left). The enhanced pathogen enrichment process is achieved via the use of dimethyl suberimidate (DMS) and ADE (Fig. 1, top middle). As an HI reagent, DMS reacts with the amine groups of ADE and generates more positively charged amidine bonds, which then directly attract negatively charged pathogens (Fig. 1, bottom left). In this case, the saline-functionalized amine groups are the basis of this all-in-one hand-held extraction approach. Fourier-transform infrared (FTIR) spectrum analysis was employed to ensure the successful amine functionalization, as shown in Fig. S1. To confirm the pathogen enrichment, we performed a fluorescence image test using 4′,6-diamidino-2-phenylindole (DAPI) staining (Fig. S2). This showed clearly fluorescent images of ADE-enriched samples. Furthermore, ADE’s nano-porous structure gives it strong absorption capability and it has an ultra-high reaction area, which also contributes to its enhanced performance^34^. A syringe filter was utilized to trap ADE and its attached pathogens, so the enrichment process could easily be performed by mixing the DMS and ADE with pathogens in solution and then injecting this onto the filter. After washing, the trapped pathogens were lysed *in situ* (Fig. 1, top right). Subsequently, the NAs were isolated chemically via a reversible crosslinking reaction.

The DMS serves as a cross linker between amine groups of NAs and ADE (Fig. 1, bottom right). This reaction is reversed at pH 10^33^. Therefore, the NAs released upon lysis are crosslinked to the surface of ADE in the presence of reaction buffer at pH 8 and can then be released by the elution buffer at pH 10. As such, it is easy to extract NA templates from a sample on the filter by controlling the pH of the injected buffers. This method is particularly effective for RNA isolation because the covalent bonds between the RNA and ADE make the RNA resistant to degradation^35^. This method does not require sample pretreatment to remove the RNase, nor any electrical apparatus such as a centrifuge, vortex, or pump, which may not be options in remote areas. This method could be readily adapted for use in a POCT system.

Clinical samples are commonly collected in relatively large volumes, varying from several to dozens of milliliters, depending on the source (e.g., tears, blood, or urine). However, given the limited capacity for sample handing, only part of the collected clinical samples is used. Here an enriched component is integrated into our proposed system since the sensitivity is naturally improved if all of the samples can be treated. Although a conventional NA extraction kit can deal with up to 15 mL of sample, its sensitivity is markedly low (Qiagen Midi and Maxi Kits, Fig. S3). In particular, large-volume extraction kits require additional equipment such as a conical tube centrifuge system. Moreover, the 15 mL extraction kit is expensive. Thus, typical kits with a sample volume capacity of 200 μL (e.g., Qiagen Mini, 200 μL volume) are more widely used in clinical diagnosis^36,37^. Therefore, to evaluate our proposed assay, we used the Qiagen Mini Kit as a standard control rather than the Qiagen Midi or Maxi Kit.

### Optimization of the extraction method

**Figure 2:**
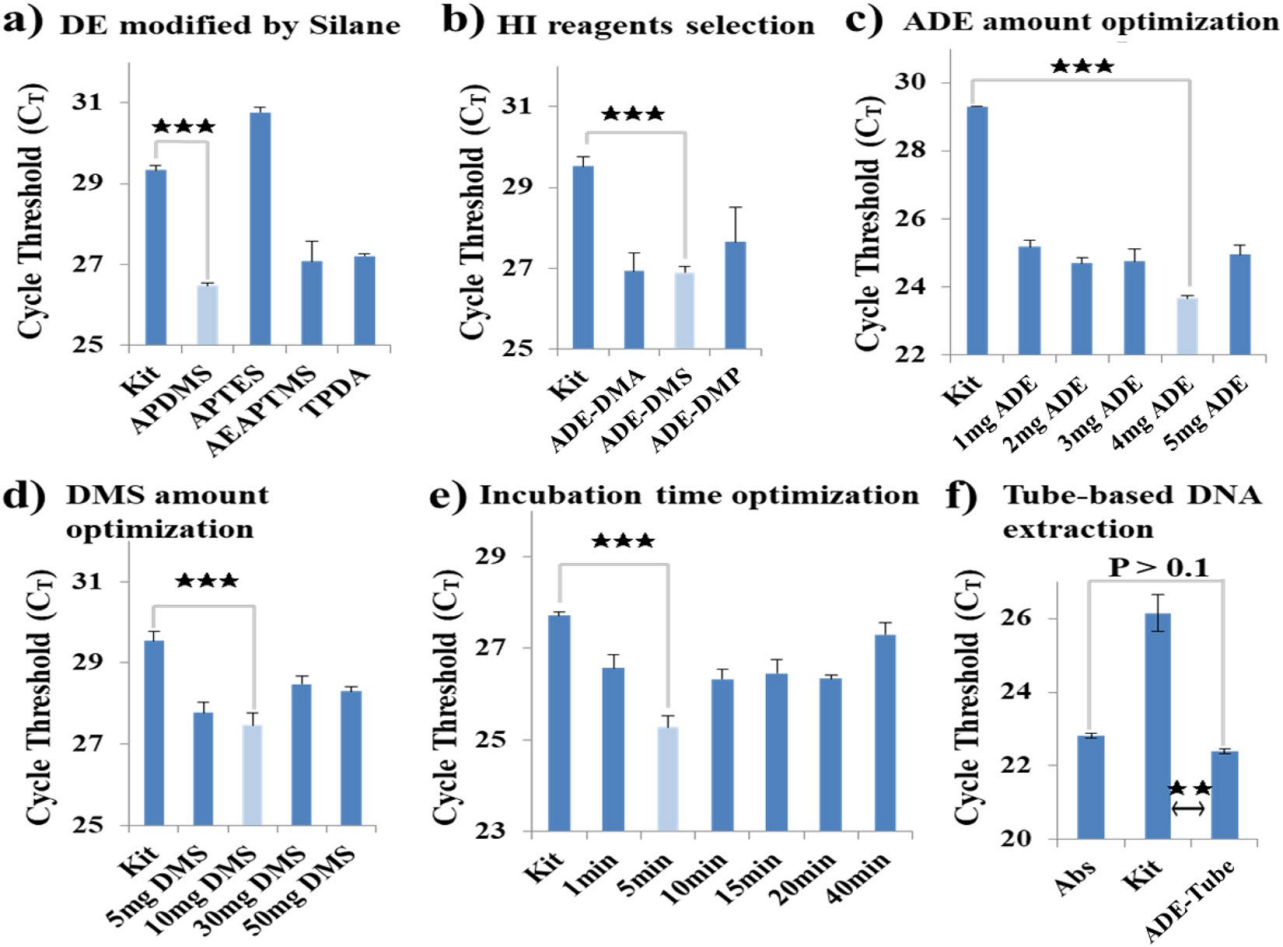
The nucleic acid (NA) isolation process was optimized and evaluated based on the cycle threshold (C_T_) values after performing real-time quantitative PCR (qPCR). (**a**–**e**) Optimization of the RNA isolation process. (**a**) Screening of different silanes for DE amine functionalization (ADE) with the following protocol: 4 mg of ADE and 20 mg of DMS incubated for 10 min, washing three times, and 1 min of elution. (**b**) Optimization of the three different homobifunctional imidoester (HI) reagents (DMA, DMS, and DMP) with the same protocol as in (**a**). (**c**) Optimization of the amount of ADE with the following protocol: 1 to 5 mg of DA with 20 mg of DMS, incubated for 10 min; washing three times, and 1 min of elution. (**d**) Optimization of the amount of DMS with the following protocol: 4 mg of DE with 5, 10, 30, 50 mg of DMS incubated for 10 min; washing three times, and 1 min of elution. (**e**) Optimization of the incubation time for RNA isolation with the following protocol: 4 mg of DE and 20 mg of DMS, incubated for 1, 5, 10, 15, 20, and 40 min; washing three times, and 1 min of elution. (**f**) Evaluation of DNA templates isolated under optimized conditions. No C_T_ values were obtained from the amplification of no-template controls (NTCs) in any of the above experiments. In all cases, the RNA was extracted from samples with the same concentration of *Brucella ovis* (10^4^ CFU/mL). For the 1 mL 10^4^ CFU/mL samples, the kit used 200 μL of the 1 mL samples whereas the tube-based assay used the entire 1 mL samples because of the difference in capacity. “Abs” refers to absolute NA references extracted from 100 μL of 10^5^ CFU/mL samples, which contained the same amount of pathogens as the 1 mL 10^4^ CFU/mL samples. All of the samples were diluted from the same stock solution. Details of the optimization process are provided in the supporting information. Error bars indicate standard deviation from the mean based on at least three independent experiments. The *p*-values were evaluated by Student’s *t*-test (^★★★^*p* < 0.001; ^★★^*p* < 0.01).

Each factor that could affect the method was optimized individually. We evaluated RNA extraction efficiency under different ADE and DMS conditions, as well as at different incubation times, using the cycle threshold (C_T_) of quantitative RT-PCR (qRT-PCR) as an indicator of quality (Fig. 2); lower C_T_ values are indicators of higher-quality NA templates^38^. All of the optimization processes used 1 mL aliquots of *Brucella ovis* samples (10^4^ CFU/mL in phosphate-buffered saline (PBS)) and the results were compared to those obtained using commercial kits (QIAamp DNA Mini Kit, RNeasy Mini Kit, RNeasy Midi Kit, and RNeasy Maxi Kit, all from Qiagen). Amine functionalization of ADE is the first step of the method, contributing to both pathogen enrichment and the NA extraction process; different silane reagents were investigated for ADE functionalization (Fig. 2a). The lowest C_T_ value was obtained after treatment with 3-aminopropyl(diethoxy)methylsilane (APDMS), which was selected for further development. Similarly, various imidoesters were tested as potential crosslinkers, and DMS was found to perform best (Fig. 2b). DMS was the only crosslinker that was stable at RT, providing additional benefits with respect to storage and transportation for future POCT^35^. Finally, the amounts of ADE and DMS were optimized with respect to pathogen enrichment (Fig. 2c,d, respectively). The second step of this method is cell lysis to facilitate the release of RNA and simultaneous crosslinking to ADE by DMS. We therefore tested the method’s performance at various incubation times and observed the highest efficiency at 5 min (Fig. 2e). The washing step was tested to check that PCR inhibitors had been removed from the NA samples. Washing twice was selected for the protocol (Fig. S4a); washing more times gave similar C_T_ values (Fig. S4a), probably due to the formation of robust covalent binding in the assay treatment process. After washing, elution buffer was added; the incubation time for elution was therefore investigated (Fig. S4b). There was no substantial improvement in efficiency at longer incubation times, suggesting that the RNA was stable in this system. It was interesting that the RNA was stable over 20 min, given that C_T_ remained constant, even though RNA should be degraded immediately in RNase-contaminated solution at RT^39^; this suggested that samples could be stored refrigerated for prolonged periods prior to analysis. The optimized system was utilized to test DNA extraction (Fig. 2f). Then, the quantity and purity of DNA isolated by the methods were measured. The purity of DNA isolated from ADE was lower than that of the kit due to the diatom contamination, which can be inhibit the optical density measuring. However, the quantity of DNA isolated from ADE was higher than that of the kit (Fig. S4c,d). Capture efficiency was evaluated by comparing C_T_ values obtained from the system with those obtained from kit-extracted 100 μL samples containing the same number of pathogens as for RNA testing (*Brucella ovis* in PBS, 10^5^ CFU/mL), which were used as absolute reference values. The kit used to obtain these reference values was the QIAamp DNA Mini Kit (200 μL), which is a typical NA isolation method that is widely used in clinical diagnosis. No C_T_ values were obtained from the amplification of no-template controls in any of the experiments. The capture efficiency of the method compared to the commercial kit reference values was 95% and 81% for DNA and RNA isolation, respectively (Fig. S5a). Finally, we integrated the ADE extraction method with a syringe filter system. After optimizing the conditions, the efficiency of the filter-based system was tested and the PCR performance of the templates from the kit and our filter-based system was compared. The templates produced using our method showed significantly better performance than those produced using the kits (p < 0.001, Fig. S5b). To validate successful target amplification, RNAs isolated by the system and the kit were confirmed by conventional PCR methods with melting curve analysis (Fig. S5c–e). Together, these tests showed that the all-in-one hand-held method for pathogen enrichment and NA extraction had been optimized for reagent types (Fig. 2a,b), amounts (Fig. 2c,d), and incubation times (Figs 2e and S3).

### Evaluation of the tube-based all-in-one approach

**Figure 3:**
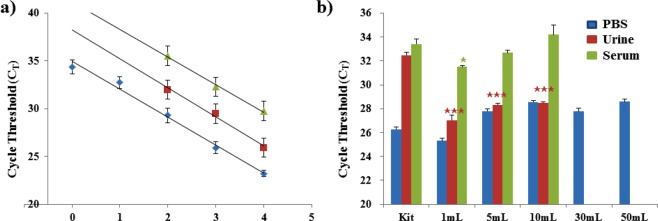
Evaluation of the ADE-tube system for RNA isolation performance based on the cycle threshold (C_T_) values of real-time quantitative PCR (qPCR). (**a**) qPCR performance of isolated RNA templates from a dilution series of pathogen samples using commercial kits (Qiagen Kit (red) and Pathogen-specific Kit (green)) and the tube-based ADE–DMS system (ADE-Tube, blue). *Brucella ovis* in PBS was used as the pathogen. (**b**) Evaluation of the ADE-Tube system for large sample volumes in various sample matrices. The total amount of pathogenic bacteria was controlled to the same level for all of the parallel experiments. Here, 1 mL samples of 10^3^ CFU/mL *Brucella ovis* were used. The kit used 200 μL of the 1 mL samples of 10^3^ CFU/mL *Brucella ovis* in PBS, urine, and serum. To evaluate the capture performance of the assay for large-volume samples, the 1 mL samples were diluted serially to volumes up to 50 mL, using matched original solutions; the urine and serum samples were tested up to 10 mL. The tube-based assay used the entire 1 mL to 50 mL samples (10^3^ CFU of *Brucella ovis* in PBS, urine, and serum) because of its different capacity. No C_T_ values were obtained from the amplification of no-template controls (NTC) in any of these experiments. Error bars indicate standard deviation from the mean based on at least three independent experiments. The *p*-values were evaluated by Student’s *t*-test (^★^*p* < 0.05, ^★★^*p* < 0.01, ^★★★^*p* < 0.001; different colors indicate the matched samples compared with the kit).

To evaluate the capability of our proposed system for on-site sample preparation, a dilution series of pathogenic *Brucella ovis* bacteria in 1 mL of PBS was prepared with concentrations of 10°–10^4^ CFU/mL. RNA templates were prepared using our method and a commercial kit, followed by qPCR analysis for pathogen detection. This showed the detection limit for our system to be 1 CFU/mL (Fig. 3a), which was 100-fold more sensitive than those of the Qiagen kit and pathogen-specific kit (10^2^ CFU/mL). Both methods produced linear relationships between C_T_ and the sample concentration (log C). The mean distance between the two fitted curves was 3.298 (Table S2), which indicated that templates extracted from the same samples were delayed by approximately 3.3 cycles when the enrichment process was not used. In a system that is 100% efficient, enriched samples should contain 10-fold more NA than samples that have not been enriched (1 mL vs. 100 μL sample volumes at the same concentration), so this result indicates that almost all of the pathogens were captured and the NAs recovered^38^. Our method can accommodate samples of up to 50 mL from various matrices, such as PBS, urine, and serum (Fig. 3b); this is because it produces RNA templates that exhibit highly effective amplification from samples produced by spiking 10 mL of the various matrices with 10^3^ CFU *Brucella ovis*. Human samples were tested to confirm there was no biologically relevant pathogen contamination (Fig. S6a,b). Compared with the absolute references by kit, there were delays of 2.3 and 0.8 cycles in the templates from 10 mL PBS and serum samples, respectively; however, clear early cycles were observed in all the templates from the urine samples. For the kit-treated samples shown in Fig. 3b, C_T_ values for the urine and serum samples were greatly delayed compared with those for the PBS samples, which may have contributed to the RNase and PCR inhibitors in the human samples^40,41^. However, pathogens showed a similar performance in the urine and PBS samples treated by assays, which suggested that the PCR inhibitors in urine were removed by the washing process involving non-precipitating NA isolation through the reversible crosslinking reaction^30^. It is notable that, for samples extracted by both kit and assay treatment, the serum samples commonly showed much longer delays than the PBS samples. The serum samples contained much more RNase, so the released RNA could easily become degraded when using the standard method; conversely, the earlier cycles observed with the assay treatment suggested that the RNA extracted by our assay benefited from covalent attachment to the matrix, inducing resistance to RNase degradation^30^. In addition, the samples diluted into 50 mL of PBS exhibited no statistically significant variations in template amplification compared with the 30 mL and 10 mL PBS samples (P > 0.1 and P > 0.5, respectively). This superior performance might be explained by the clean buffer solution, which contained no other cells or proteins. We further tested our method with other pathogens, including *Salmonella enterica* and *Aspergillus fumigatus*. PCR results from the extracted templates showed a good level of enrichment for *Salmonella enterica* (Fig. S6c), but PCR results from *Aspergillus fumigatus* from ADE-filter and fungal kit methods showed no Ct value due to the difficulty in breaking the outer membrane of the fungi (Fig. S6d). Although further investigation would be needed for fungi with a thick outer membrane, our method confers substantial improvement in pathogen detection limits for low-concentration samples, particularly those with a large volume.

### Performance of the all-in-one hand-held method

**Figure 4:**
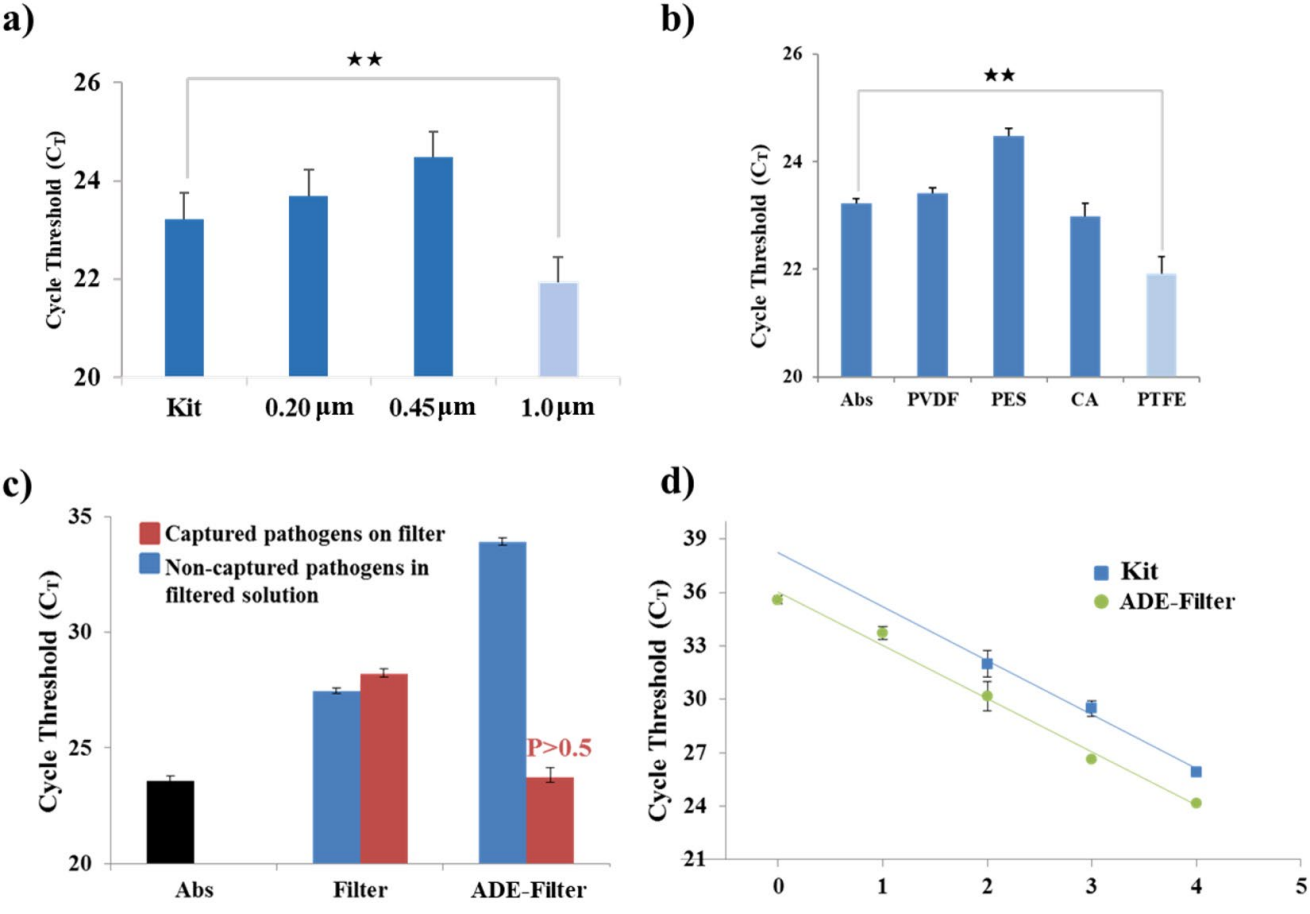
Evaluation of the ADE-filter system for RNA isolation based on the cycle threshold (C_T_) values of real-time quantitative PCR (qPCR), showing the effect of common syringe filter materials on the ADE-filter system’s RNA isolation efficiency. (**a**) The investigation of filter membranes included (**a**) different pore sizes (0.2, 0.45, and 1 μm) and (**b**) different materials, including cellulose acetate (CA), polyethersulfone (PES), polyvinylidene fluoride (PVDF), and polytetrafluoroethylene (PTFE). All of the RNA templates were isolated from a sample containing 10^5^ CFU/mL of *Brucella ovis* in PBS. (**c**) Comparison of a naked filter with the proposed ADE–DMS-assisted filter system for producing RNA templates from pathogens captured on a filter and from non-captured pathogens in solution. Captured pathogens on filter refers to tested samples being subjected to the assay following the protocol described in the section “Filter-based pathogen enrichment and NA extraction.” NA was isolated from the pathogens captured by the assay. The non-captured pathogens in filtered solutions indicated that NA was isolated from the remaining solutions after the assay treatment. NA was isolated from the pathogens not captured by the assay. “Abs” refers to absolute NA references extracted from 100 μL of 10^6^ CFU/mL samples, which contained the same amount of pathogens as the 1 mL of 10^5^ CFU/mL samples. All of the samples were diluted from the same stock solution. (**d**) The qPCR performance of isolated RNA templates from both a commercial kit (Kit) and the syringe-based ADE system (ADE-Filter) using *Brucella ovis* in PBS. No C_T_ values were obtained from the amplification of no-template controls (NTC) in any of these experiments. Error bars indicate the standard deviation from the mean based on at least three independent experiments. The *p*-values were evaluated by Student’s *t*-test (^★^*p* < 0.05, ^★★^*p* < 0.01, ^★★★^*p* < 0.001; different colors indicate the matched samples compared with the kit).

**Figure 5:**
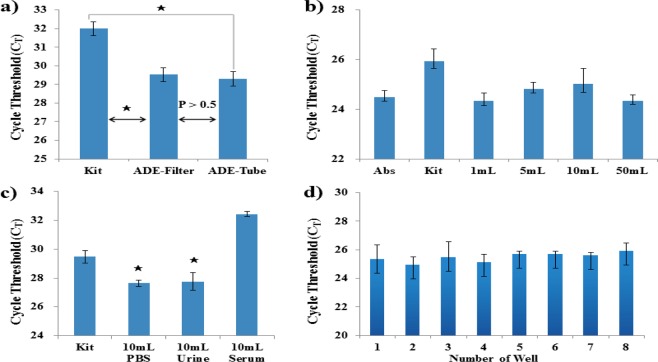
Performance evaluation of the ADE-filter system for large sample volumes in various sample matrices. The total amount of pathogenic bacteria was controlled at the same sample level for all of the parallel experiments. (**a**) Performance of the ADE–DMS system in both tube and filter formats. Here, 1 mL of 10^2^ CFU/mL *Brucella ovis* in PBS samples were used. The RNA isolation process followed that described in the sections “Tube-based pathogen enrichment and nucleic acid extraction” and “Filter-based pathogen enrichment and nucleic acid extraction.” Error bars indicate the standard deviation from the mean based on at least three independent experiments. The *p*-values were evaluated by Student’s *t*-test (^★^*p* < 0.05). (**b**) RNA isolated by the ADE-filter system from different volume samples (1 mL to 50 mL) containing 10^4^ CFU of *Brucella ovis* in PBS. For the 1 mL (10^4^ CFU/mL) samples, the kit used 200 μL of the 1 mL samples whereas the filter-based assay used the entire 1 mL samples because of its different capacity. “Abs” refers to the absolute NA references extracted from 100 μL (10^5^ CFU/mL) samples, which contained the same amount of pathogens as the 1 mL of 10^4^ CFU/mL samples. All of the samples were diluted from the same stock solution. (**c**) RNA isolated by the ADE-filter system from different sample matrices containing 10^3^ CFU of *Brucella ovis* in all of the 10 mL PBS, urine, and serum samples. No C_T_ values were obtained from the amplification of no-template controls (NTC) in any of these experiments. Error bars indicate standard deviation from the mean based on at least three independent experiments. The *p*-values were evaluated by Student’s *t*-test (^★^*p* < 0.05, ^★★^*p* < 0.01; different colors indicate the matched samples compared with the kit). (**d**) Robust testing with 96-well filter plates. DNA isolated by the ADE 96-well filter plate from the sample containing 10^3^ CFU of *Brucella ovis* in the 1 mL PBS sample. Eight samples were treated simultaneously. Error bars indicate standard deviation from the mean based on at least three independent experiments.

To achieve complete laboratory independence, we integrated our method into a syringe filter system. This method was powered entirely by hand via a syringe and needed no other devices. Furthermore, because it benefited from our RNA extraction/stabilization strategy, there was no need to operate the whole system in an RNase-free environment. This means that our sample preparation method was perfectly suited to POCT applications, especially for the application in resource-limited settings, owing to its low-cost, as well as ease of use and production. Using optimized conditions, we tested several commercial filters; several types of filter and pore sizes produced enrichment (Fig. 4a,b). The earliest C_T_ cycle clearly resulted from using the PTFE filter, the filter with the highest alkali stability and lowest protein binding capacity. RNA templates produced using a naked filter were compared with those produced using our filter containing the ADE and DMS system (Fig. 4c), for both enriched samples (captured pathogens eluted from filters) and non-enriched samples (non-captured pathogens in the filtered solution). Without the assistance of ADE and DMS, the PTFE filter was not suitable for pathogen enrichment and RNA extraction because some cycle delay was observed with the enriched samples relative to the non-enriched samples, possibly due to the loss of some solvent or bacteria on the filter. In contrast, our method using a filter with ADE and DMS resulted in high enrichment and 98.3% recovery of pathogens compared to the commercial reference value. We further tested the performance of this hand-held syringe method in the preparation of RNA templates from a dilution series of pathogens, assessing the performance using qPCR (Fig. 4d). The performance of the syringe method was comparable to that of the tube-based method, resulting in an ultra-low detection limit of 10^0^ CFU/mL, which was 100-fold better than that of the kit system (10^2^ CFU/mL). There was no significant difference in pathogen capture efficiency between tube-based and filter-based assays (Fig. 5a). The capture efficiency of the tube-based assay is 99.2%, calculated by the C_T_ values, as mentioned above. Since the filter-based assay presents 98.3% capture efficiency, 0.9% of pathogens were lost via changing the platform from tube to filter. However, both systems exhibited high efficiency in pathogen enrichment and NA extraction, suggesting that the proposed method could easily be integrated with other assays to produce high-quality RNA templates on-site. However, the tube-based assay requires a centrifugal device whereas the filter-based system does not require any specialized instruments and can be completed within 20 min, which is much faster than the tube-based system. In addition, the filter-based method can easily be used with large sample volumes because of the easy loading step using the syringe, allowing samples of up to 50 mL to be processed easily. The enrichment rates of samples with different pumping volumes were calculated based on the Ct values in Fig. 5b, shown as 99.8%, 79.0%, 68.8%, and 95.9% for 1 mL, 5 mL, 10 mL, and 50 mL samples, respectively. Our assay also delivered effective enrichment and on-site NA extraction with samples of various volumes (Fig. 5b), as well as exhibiting relatively stable performance. We also tested the performance of the method with other biological matrices and found that it performed equally well with urine samples, but less well with serum samples, for which greater variability was observed; however, the difference was not statistically significant (Fig. 5c). Next, we applied ADE to a 96-well filter plate for robust sample preparation. When the eight samples were simultaneously treated using the ADE 96-well filter plate, the DNAs were isolated and amplified with good uniformity (Fig. 5d). Furthermore, we used FTA card as an instrument-free technique for NA sample preparation. However, the detection limit of the FTA card for both RNA (10^5^ CFU) and DNA (10^3^ CFU) was less sensitive than that of our assay (Fig. S6e).

## Conclusions

We have developed a universal protocol for pathogen enrichment and on-site NA sample preparation using a simple, all-in-one, filter-based system. Using a common syringe, NA templates were successfully extracted without any laboratory support. To evaluate the method’s efficiency, NA templates from replicate extractions were analyzed by qPCR. The new method was found to be suitable for NAT-based pathogen diagnosis, producing an ultra-low detection limit of 10^0^ CFU/mL in urine samples, which was 100-fold better than that of commercial kits. There are several instrument-free techniques that were reported previously^24,42–44^. Among these, paper-based assays like FTA cards have attracted increasing attention since they are low-cost and easy to fabricate. Endow with natural porous assay, it is easy to achieve sample preparation, storage, extraction, and concentration using a paper-based assay^24^. However, owing to paper’s intrinsic properties, the reuse of paper-based assays is generally difficult. In addition, FTA card-based assays are usually designed for small-volume samples (on the order of microliters), but not suitable for large-volume samples (on the order of milliliters)^43^. Compared with other systems, our proposed filter-based method provides several benefits, including high sensitivity and specificity, low cost, ease of manufacture and operation, no need for specialized equipment, and universality of use (Table 1). In addition, we demonstrated the method’s ability to process large sample volumes, of up to 50 mL, with a variety of biological matrices. Because all of the reagents used are stable at RT, the proposed assay should be amenable to storage and transportation without a cold chain. Further improvements to allow the processing of more complex clinical samples such as whole blood are still required, to fully realize the integration of this method into NAT analysis systems. Nevertheless, the proposed protocol exhibits great potential for use in NAT-based POCT applications, especially in resource-limited settings, due to its high performance.

**Table 1:** Advantages and disadvantages of different NA isolation methods.

Items	ADE-filter	Reported single-tube system^30^	Column-based kit^45^	FTA-based assay^43^	Chip-based assay^44^
Electrical power	Not needed	Partly; battery-driven centrifugal device is needed	Needed	Partly; battery-driven heater is needed	Partly; battery-driven heater is needed
Pathogen enrichment	Yes	Yes	No	Partly	No
Total time	Under 20 min	60 min	20–30 min for extraction process only	75 min	60 min
Other instruments	Not needed	Centrifugal device needed	Large centrifuge and thermal incubator needed	Not needed	Partly; portable heater
Cost	Low	Low	High	Low	Cost-effective
Sensitivity	10^0^ CFU/mL	10^0^ CFU/mL	10^2^ CFU/mL	3.0 × 10^0^ CFU	3.0 × 10^0^ copies
Specificity	High	High	High	High	High
Reproducibility	High	High	High	High	High
Thermal control	Not needed	Needed	Needed	Not needed	Needed
Surroundings	Household	Laboratory-based	Laboratory-based	Household	Laboratory-based
Complexity of integration with another assay	Easy	Difficult	Impossible	Easy	Easy

## Methods

### Chemicals and reagents

DE (calcined powder), acetic acid (99.7%), dimethyl suberimidate dihydrochloride (DMS, 98%), lysozyme solution (30 mg/mL in distilled water), (3-aminopropyl) triethoxysilane (APTES, 99%), 3-aminopropyl(diethoxy)methylsilane (APDMS, 97%), [3-(2-aminoethylamino) propyl] trimethoxysilane (AEAPTMS, 80%), and N1-(3-trimethoxysilylpropyl) diethylenetriamine (TPDA, technical grade) were purchased from Sigma-Aldrich, St. Louis, MO, USA. Ethanol (99%) and phosphate-buffered saline (PBS, 10×, pH 7.4) were ordered from Thermo Fisher Scientific, Waltham, MA, USA. DNase solution (1500 Kunitz units RNase-free DNase I) and Proteinase K solution (>600 mAU/mL) were purchased from Qiagen, Germany. Brucella agar was purchased from MB cell, Seoul, Korea. 5% defibrinated sheep blood was purchased from Kisan Bio, Seoul, Korea. nutrient broth and trypticase soy broth were purchased from BD Difco, Sparks, MD, USA. Sabouraud dextrose agar with chloramphenicol was purchased from Hardy Diagnostics, Santa Maria, CA, USA. All the regents were used without any further purification.

### Preparation of amine-functionalized diatomaceous earth

Amine-functionalized diatomaceous earth (ADE) was utilized as the matrix in both the enrichment and extraction processes and was prepared as follows. DE was washed with distilled water (DW) for 30 min with vigorous stirring. The sediment containing impurities was removed after a short period of settling under gravity. Four types of silanes (APTES, APDMS, AEAPTMS, and TPDA) were used to prepare the ADE. Briefly, 5 mL of silane was pipetted dropwise into 100 mL 95% (v/v) ethanol solution, which was acidified with acetic acid (pH 5). Then, 2 g diatomaceous earth (DE) was added with vigorous stirring. The reaction was maintained at room temperature (RT) for 4 h. The ADE was washed with ethanol and then dried under vacuum overnight, and the dried ADE was stored at RT until further analysis.

### Cell culture

*Brucella ovis* (ATCC 25840) was used to evaluate the pathogen diagnostic method. *Brucella ovis* was grown in Brucella agar containing 5% defibrinated sheep blood and incubated at 37 °C in an atmosphere of 5% CO_2_ for 48 h. *Escherichia coli* (ATCC 25922) was inoculated in nutrient broth medium and incubated overnight at 37 °C under shaking conditions. *Salmonella enterica* (ATCC 14028) was inoculated into trypticase soy broth and incubated for 24 h at 37 °C under an aerobic atmosphere. After culturing, the bacterial suspension was quantified by the agar plate method and subsequently diluted to different concentrations in PBS. *Aspergillus fumigatus* (ATCC 36607) was grown in Sabouraud dextrose agar with chloramphenicol at 25 °C for 5 days. After culturing, the *Aspergillus fumigatus* re-suspended in PBS and quantified by the hemocytometer.

### Tube-based pathogen enrichment and NA extraction

ADE and DMS were used as the enrichment and extraction matrices for PBS, urine, and serum samples. The urine samples were collected daily. The human serum sample was purchased from Sigma-Aldrich (H4522-100ML). First, 80 μL of ADE suspension (50 mg/mL in DW) and 100 μL of DMS solution (100 mg/mL in DW) were pipetted into a sample solution. With the 1 mL samples, the pathogens were collected with ADE after 1 min of settling under gravity at RT, whereas the 50 mL samples were incubated on a rotating mixer (Topscien Instrument Co., Ltd., Ningbo, China) for 30 min at 99 rpm. After washing with 1 mL of PBS, the enriched pathogens were harvested by centrifugation. NA isolation was subsequently performed in the same tube. Briefly, 20 μL of Proteinase K, 150 μL of in-house lysis buffer (100 mM Tris-HCl [pH 8.0], 10 mM ethylenediaminetetraacetic acid, 1% sodium dodecyl sulfate, and 10% Triton X-100), 30 μL of lysozyme solution (30 mg/mL in DW), and 10 μL of RNase-Free DNase solution were added separately. After mixing, the tube was incubated in a thermal shaker at 850 rpm either for 30 min at 56 °C for DNA extraction or for 10 min at RT for RNA extraction. NA templates from lysed cells were immobilized on ADE through DMS crosslinking. The supernatant was removed with a short centrifugal pulse and the pellet was washed twice with 200 μL of PBS. For reverse crosslinking, 100 μL of elution buffer (10 mM sodium bicarbonate, pH > 10, adjusted by NaOH) was added and incubated for 1 min at RT. After brief centrifugation, the supernatant containing the isolated DNA or RNA was removed and stored at −20 °C until needed. As a positive control, the same samples were subjected to NA extraction using commercial kits (QIAamp DNA Mini Kit and RNeasy Mini Kit, Qiagen) following the manufacturer’s protocols. The maximum capacity of these kits was considered to be 200 μL of a 1 mL sample.

### Filter-based pathogen enrichment and NA extraction

As with the tube-based procedure, 80 μL of ADE suspension (50 mg/mL in DW) and 100 μL of DMS solution (100 mg/mL in DW) were pipetted into a sample solution. With the 1 mL samples, the mixture was loaded into a 5 mL syringe and inverted twice. No extra incubation time was needed. An incubation time of 30 min was used for larger volume samples (5 to 50 mL). During incubation, the mixture was occasionally shaken by hand to disperse the sample. For the 50 mL samples as an alternative option, a rotating mixer (Topscien Instrument Co., Ltd., Ningbo, China) set to 99 rpm was used, with incubation for 30 min. After incubation, the mixture was passed through a polytetrafluoroethylene (PTFE) syringe filter with 1.0 μm pores (Whatman, USA) and then washed with 1 mL of PBS, also using the syringe. The remaining solution in the filter unit was removed by pumping air through it with the syringe; this was repeated twice. NAs were extracted from the bacteria trapped on the filter by injecting a mixture of 20 μL of Proteinase K, 150 μL of in-house lysis buffer, 30 μL of lysozyme solution, and 10 μL of RNase-Free DNase solution. The mixture could fill most of the filter. Then, the filter was inverted and incubated either for 30 min at 56 °C for DNA or for 10 min at RT for RNA extraction. An empty syringe was used to pump air into the filter and the mixture was naturally removed from the filter. The filter was then washed twice with 1 mL of PBS followed by air. Finally, 100 μL of elution buffer was injected onto the filter and, after 1 min of incubation at RT, the elution buffer containing NAs was collected by pumping air and stored at −20 °C until needed. It should be noted that the use of diethylpyrocarbonate-treated water is not recommended for this method because it may disturb the reversible crosslinking-based RNA isolation process, and because the covalent bonds between the RNA and ADE make the RNA stable and resistant to the RNase. For robust testing, the 96-well filter plate was combined with ADE. The plates were purchased at Merck (MultiScreen Solvinert 96-Well Filter Plate; Merck, Darmstadt, Germany).

### Conventional and real-time PCR

We performed PCR and real-time PCR to determine the quality of the isolated DNA and reverse transcription PCR (RT-PCR) and real-time RT-PCR to determine the quality of the extracted RNA. The primers used are listed in Table S1. The PCR cycling conditions were as follows: an initial denaturation step at 95 °C for 15 min; 40 cycles at 95 °C for 30 s, 58 °C for 30 s, and 72 °C for 30 s; and a final extension step at 72 °C for 7 min. Then, 5 µL of DNA was amplified in a total volume of 25 μL containing PCR buffer (10×, Qiagen), 2.5 mM MgCl_2_, 0.25 mM deoxynucleotide triphosphate, 25 pM of each primer, one unit of Taq DNA polymerase (Qiagen), and deionized (DI) water. Next, 5 μL of isolated RNA was amplified in a total volume of 25 μL containing One-step RT-PCR buffer (5×, Qiagen), 0.25 mM deoxynucleotide triphosphate, 25 pM of each primer, 1 μL of RT-PCR One-step enzyme mix (Qiagen), and DI water. The following thermal profile was used for RT-PCR: 30 min reverse transcription at 50 °C; 15 min pre-denaturation at 95 °C; 40 cycles of 30 s at 95 °C, 30 s at 58 °C, and 30 s at 72 °C; and a final extension at 72 °C for 10 min. The PCR and RT-PCR products were separated by gel electrophoresis on 2% agarose gels containing ethidium bromide (EtBr) and imaged with a Gel-Doc System (Clinx Science Instruments, Shanghai, China). The real-time PCR and real-time RT-PCR procedures were modified from the AriaMx real-time PCR Instrument protocol (Agilent Technologies, Santa Clara, CA, USA). Briefly, 5 µL of isolated DNA was amplified in a total volume of 20 μL containing 2× Brilliant III SYBR Green QPCR master mix, 25 pM of each primer, and DI water. An initial pre-denaturation at 95 °C for 10 min was followed by 40 cycles at 95 °C for 10 s, 58 °C for 20 s, and 72 °C for 20 s, and then by a cooling step at 40 °C for 30 s. For the real-time RT-PCR, 5 μL of isolated RNA was amplified in a total volume of 20 μL containing 2× Brilliant III SYBR Green QRT-PCR master mix, 25 pM of each primer, 0.2 mM of dithiothreitol, 1 μL of RT/RNase block, and DI water. The following thermal profile was used for the real-time RT-PCR: 20 min reverse transcription at 50 °C; 15 min pre-denaturation at 95 °C; 40 cycles of 10 s at 95 °C, 20 s at 58 °C, and 20 s at 72 °C; and a cooling step at 40 °C for 30 s. The SYBR Green signals of the amplified products were acquired using AriaMx real-time PCR (Agilent Technologies).

## Supplementary information

supplementary file
