# Supplementary material for: A robust, hand-powered, instrument-free sample preparation system for point-of-care pathogen detection
Source: Sci Rep. 2019 Nov 8;9:16374. doi: 10.1038/s41598-019-52922-y (PMC6841715; doi:10.1038/s41598-019-52922-y)
Supplement: Supplementary file 1 — supplementary file [file 41598_2019_52922_MOESM1_ESM.docx]

**Supporting Information**

A robust, hand-powered, instrument-free sample preparation system for point-of-care pathogen detection

Fei Zhao^1,2,†^, Eun Yeong Lee^1,†^, Geun Su Noh^1,†^, Jaehyup Shin^1^, Huifang Liu^1^, Zhen Qiao^1^, and Yong Shin^1,^*

^1^Department of Convergence Medicine, Asan Medical Institute of Convergence Science and Technology, Asan Medical Center, University of Ulsan College of Medicine, Seoul, Republic of Korea

^2^Academy of Medical Engineering and Translational Medicine, Tianjin University, Tianjin Key Laboratory of Brain Science and Neural Engineering, Tianjin, China

*Corresponding author: [shinyongno1@gmail.com](mailto:shinyongno1@gmail.com)

^†^These authors contributed equally to this work.

**Supporting Information**

Figure S1-6

Table S1-2

**DAPI experiment**

The nuclei of *E. coli* were stained with 4′,6-diamidino-2-phenylindole (DAPI; Thermo Fisher Scientific), following the manufacturer’s instructions. Briefly, 1 mL samples of 10^4^ CFU/mL *E. coli* were washed with PBS and centrifuged at 8000×g for 1 min. The resulting cell pellets were resuspended in 200 μL of freshly prepared DAPI staining solution at a final concentration of 300 nM. The cells were then incubated for 3 min in a box protected from light at RT. The staining solution was removed, and the cells were washed with PBS and again resuspended in 200 μL of PBS. The DAPI-stained samples were enriched using ADE and DMS, as described in the section about tube-based pathogen enrichment and nucleic acid extraction. The enrichment of *E*. *coli* on ADE was observed through a 100× oil immersion lens (Plan-Neofluar 100×/1.30; Carl Zeiss) on a fluorescence microscope (Axioskop 2; Carl Zeiss) equipped with a 100 W mercury lamp and AxioCam MRc (Carl Zeiss). Images were recorded and processed using the accompanying software ZEN 2.5 (Blue Edition, Carl Zeiss).

**Commercialized kit experiments**

For comparison, RNAprotect®Bacteria Reagent and RNeasy®Protect Bacteria Kits were purchased from Qiagen (Germany). The steps were performed following the manufacturer’s instructions. The RNAprotect®Bacteria Reagent (2 volumes) was added to 1 volume of bacterial suspension. The bacteria were broken enzymatically and by Proteinase K digestion along with centrifugation and vortexing. After the lysis and purification steps, RNA was eluted to 100 μL.

For DNA and RNA isolation as an instrument-free technique, Whatman™ FTA™ cards were purchased from GE Healthcare (USA). The steps were performed following the manufacturer’s instructions. Bacterial suspension (40 μL) was applied to each FTA Micro Card. Then, the cards were dried for at least 2 h at RT. A disposable punch was used to remove a sample card from the center of dried cards. Each card was lysed with the lysis buffer, and DNA or RNA was isolated followed by purification and elution steps.


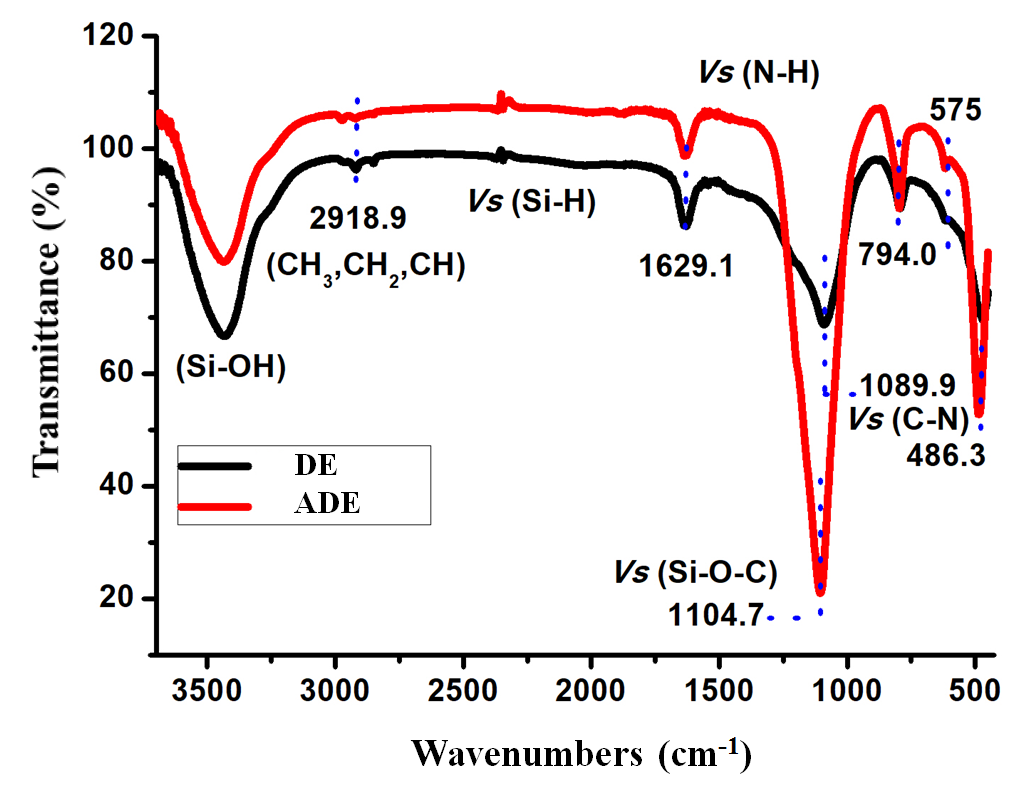


**Figure S1.** Fourier-transform infrared (FTIR) spectrum analysis of diatomaceous earth (DE) and amine-functionalized diatomaceous earth (ADE). The absorption peaks at 3295 and 2920 cm^−1^ can be attributed to Si–OH and O–H bonding on the surface of the basement DE, respectively. The absorption peaks at 1629.1 and 1089.9 cm^−1^ were attributed to asymmetric stretching vibrations in the Si–O–Si bonds, the Si–CH_2_ bond, and the C-N bond (black and red curves). After the amine functionalization, the well-defined absorption bands at 1104.7 (Si–O–C bonding), 1410–1450 (N-H bonding), and 2250.5 cm^−1^ (Si–H bonding) confirmed the amino modification.


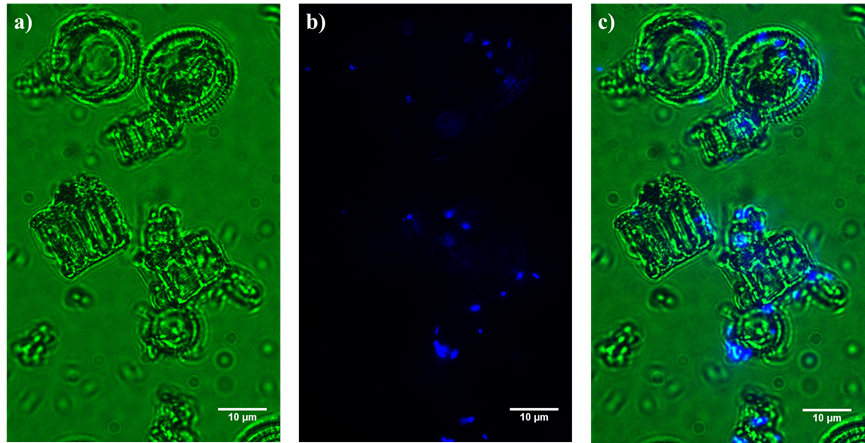


**Figure S2.** Fluorescence microscopic images of *Escherichia coli* enrichment on the surface of amine-functionalized diatomaceous earth (ADE). (a) Normal differential interference contrast image, (b) stained with 4′,6-diamidino-2-phenylindole (DAPI), and (c) a merged image.


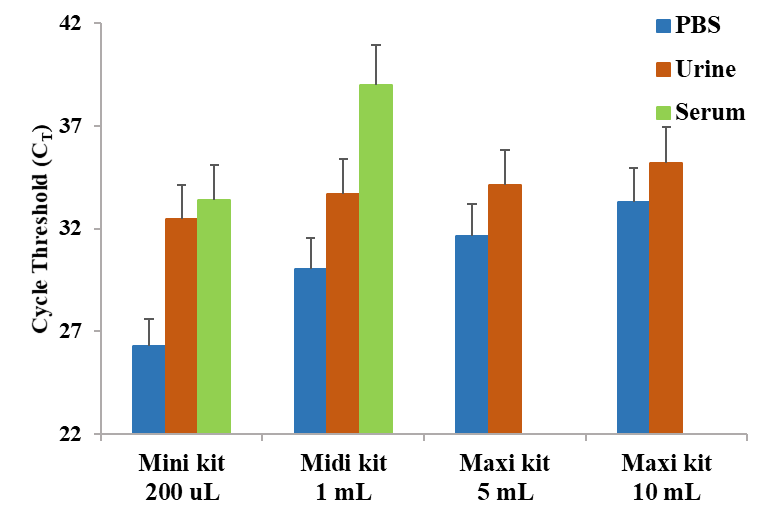


**Figure S3.** Test of the performance of commercial kits for the treatment of large-volume samples. Qiagen Mini, Midi, and Maxi kits were used. The test used 1 mL samples of 10^3^ CFU/mL *Brucella ovis* in PBS, urine, and serum diluted to 5 and 10 mL using matched original solutions. On the *x*-axis, “Kit” refers to the RNA isolated from 200 μL of 1 mL samples using Qiagen Mini kits; “1 mL” refers to RNA isolated from the entire 1 mL samples using Qiagen Midi kits; and “5 mL” and “10 mL” indicate the RNA isolated from entire 5 and 10 mL samples using Qiagen Maxi kits. All of the samples were diluted from the same stock solution. No C_T_ values were obtained from the amplification of no-template controls (NTC) in any of these experiments. Error bars indicate standard deviations from the mean based on at least three independent experiments.


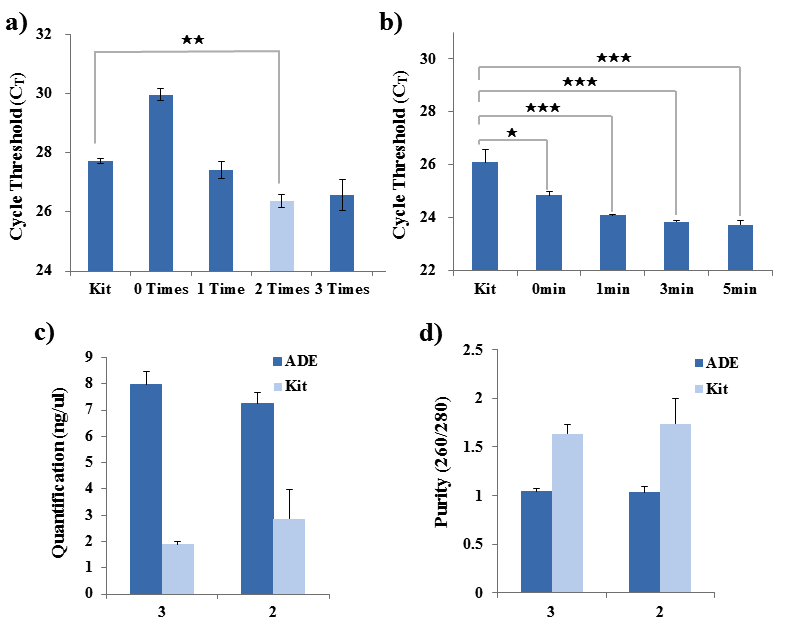


**Figure S4.** The characterization of the nucleic acids isolated. (a, b) Optimization of the washing and elution step. This used 1 mL samples of 10^4^ CFU/mL *Brucella ovis* in PBS. “Kit” refers to the standard protocol treatment with the Qiagen Kit (which has a 200 μL capacity, so only 200 μL samples from 1 mL of 10^4^ CFU/mL sample could be treated). “Others” on the *x*-axis indicates the performance of the proposed assay (which has a large-volume capacity, so the entire 1 mL 10^4^ CFU/mL samples could be treated). Other *x*-axis captions indicate the washing (a) or elution times (b) in the NA isolation protocol, as described in the section “Tube-based pathogen enrichment and nucleic acid extraction.” Error bars indicate standard deviations from the mean based on at least three independent experiments. The *p*-values were evaluated by Student’s *t*-test (★★★: *p* < 0.001; ★★: *p* < 0.01; ★: *p* < 0.05). (c-d) Comparison of quantity and purity of the DNA isolated from ADE and kit methods. Error bars indicate standard deviations from the mean based on at least three independent experiments.


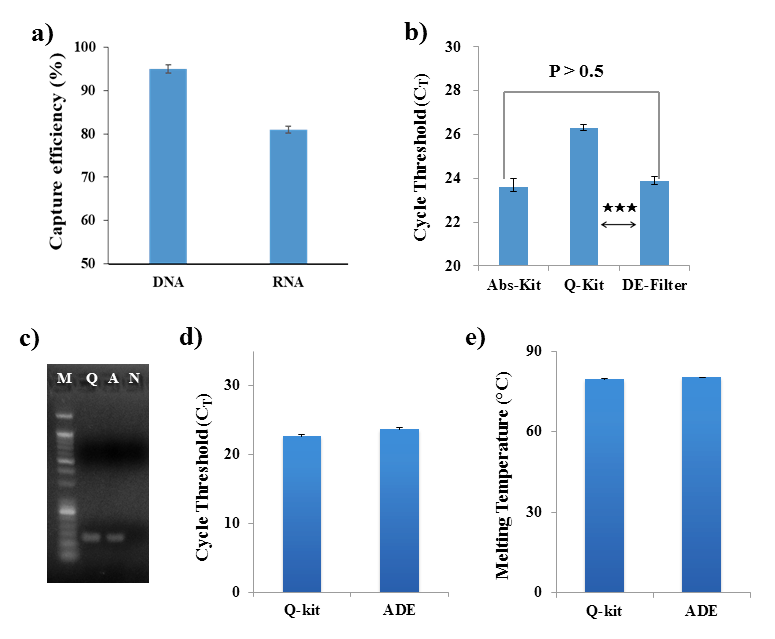


**Figure S5.** Capture efficiency of the optimized tube-based assay during DNA and RNA extraction. (a) The capture efficiency was evaluated by comparing the assay extraction with the absolute reference level of extraction by a widely used kit with the same concentration of pathogens. The same final amount of 10^4^ CFU pathogen was used in each case: the ADE assay used 1 mL of 10^4^ CFU/mL samples whereas the absolute reference used 100 μL of 10^5^ CFU/mL samples. All of the samples were diluted from the same stock solution. Error bars indicate standard deviations from the mean based on at least three independent experiments. (b) Quality check of the RNA templates isolated by the kit and filter. Here, 1 mL samples of 10^4^ CFU/mL *Brucella ovis* in PBS were tested by the kit and using our assay. “Abs-Kit” refers to the absolute reference samples extracted by the kit (100 μL of 10^5^ CFU/mL, making 10^4^ CFU in total); “Q-Kit” refers to the standard protocol treatment using the Qiagen Kit (with a 200 μL capacity, so only 200 μL samples from the 1 mL of 10^4^ CFU/mL sample could be treated); “ADE-Filter” refers to the performance of our assay (which has a large-volume capacity, so the entire 1 mL of 10^4^ CFU/mL sample could be treated). Error bars indicate standard deviations from the mean based on at least three independent experiments. The *p*-values were evaluated by Student’s *t*-test (★★★: *p* < 0.001). (c–e) RNAs isolated from the ADE and the kits were confirmed by end-point PCR and real-time PCR with melting curve analysis. Uncropped gels are displayed.


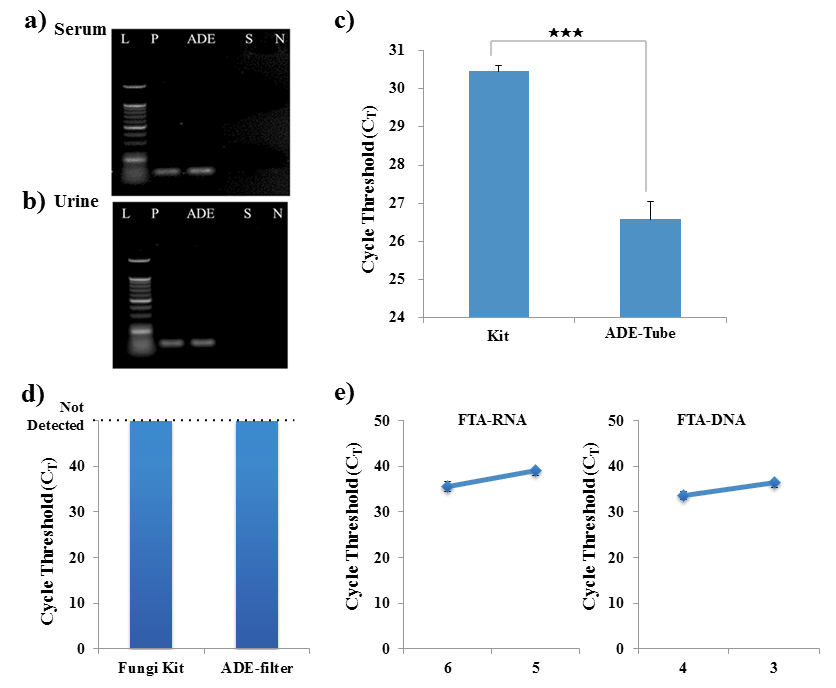


**Figure S6.** Gel electrophoresis of RT-PCR products using RNA isolated from biological fluids using a commercial kit (Kit) and the ADE-tube system, using 10^5^ CFU *Brucella ovis* samples. (a) RNA isolated from human serum samples. L, ladder; P, RNA isolated by the kit from 10^5^ CFU *Brucella ovis* in serum samples; ADE, RNA isolated by our assay from 10^5^ CFU *Brucella ovis* in serum samples; S, RNA isolated by the kit from original serum samples (not pathogen spiked); N, no-template control (NTC). (b) RNA isolated from human urine samples. L, ladder; P, RNA isolated by the kit from 10^5^ *Brucella ovis* in urine samples; ADE, RNA isolated by our assay from 10^5^ CFU *Brucella ovis* in urine samples; S, RNA isolated by the kit from original urine samples (not pathogen spiked); N, NTC. All of the RNA templates were amplified with *Brucella ovis* primers, following the protocol described in the section “Conventional and real-time PCR.” Uncropped gels are displayed. (c) The assay performance was tested using 1 mL samples of 10^6^ CFU/mL *Salmonella enterica* in PBS. The NA isolation process followed that described in the section “Tube-based pathogen enrichment and nucleic acid extraction.” Error bars indicate standard deviations from the mean based on at least three independent experiments. The *p*-values were evaluated by Student’s *t*-test (★★★: *p* < 0.001). (d) DNAs isolated from the cultured *Aspergillus fumigatus* samples (10^3^ CFU/mL) using fungal commercialized kit and ADE-filter were amplified by real-time PCR. (e) RNA (left) and DNA (right) isolated by our assay from 10^6^ (6) to 10^5^ (5) CFU/mL of *Brucella ovis* (for RNA) and 10^4^ (4) to 10^3^ (3) CFU/mL of *Brucella ovis* (for DNA), respectively, which were amplified by real-time PCR. Error bars indicate standard deviations from the mean based on at least three independent experiments.

**Table S1.** Primers’ sets for qPCR and PCR.

| **Samples** | **Targets** | **Seuqence (5’ → 3’)** |
| --- | --- | --- |
| *B. ovis* | *IS711* | F: GCTTGAAGCTTGCGGACAGT |
|  |  | R: GGCCTACCGCTGCGAAT |
| *S. enterica* | *invA* | F: TATCGCCACGTTCGGGCAA |
|  |  | R: TCGCACCGTCAAAGGAACC |
| *A. fumigatus* | *Afumi* | F: CGTGTCTATCGTACCTTGTTGCTT |
|  |  | R: AACTCAGACTGCTACTTTCAGAACAG |

**Table S2.** Constants of linear fitting functions for the performance of the all-in-one approach in the tube format.

| **Fitting curve** | **R^2^** | **Fitting function** | **Average Distance** |
| --- | --- | --- | --- |
| Kit | 0.9904 | f(x) = -3.035x + 38.236 | 3.298±0.058 |
| ADE-Tube | 0.9884 | f(x) = -2.911x + 34.925 |  |
